# Supplementary material for: Mechanism of anion exchange and small-molecule inhibition of pendrin
Source: Nat Commun. 2024 Jan 6;15:346. doi: 10.1038/s41467-023-44612-1 (PMC10771415; doi:10.1038/s41467-023-44612-1)
Supplement: Supplementary file 1 — Supplementary Information [file 41467_2023_44612_MOESM1_ESM.pdf]

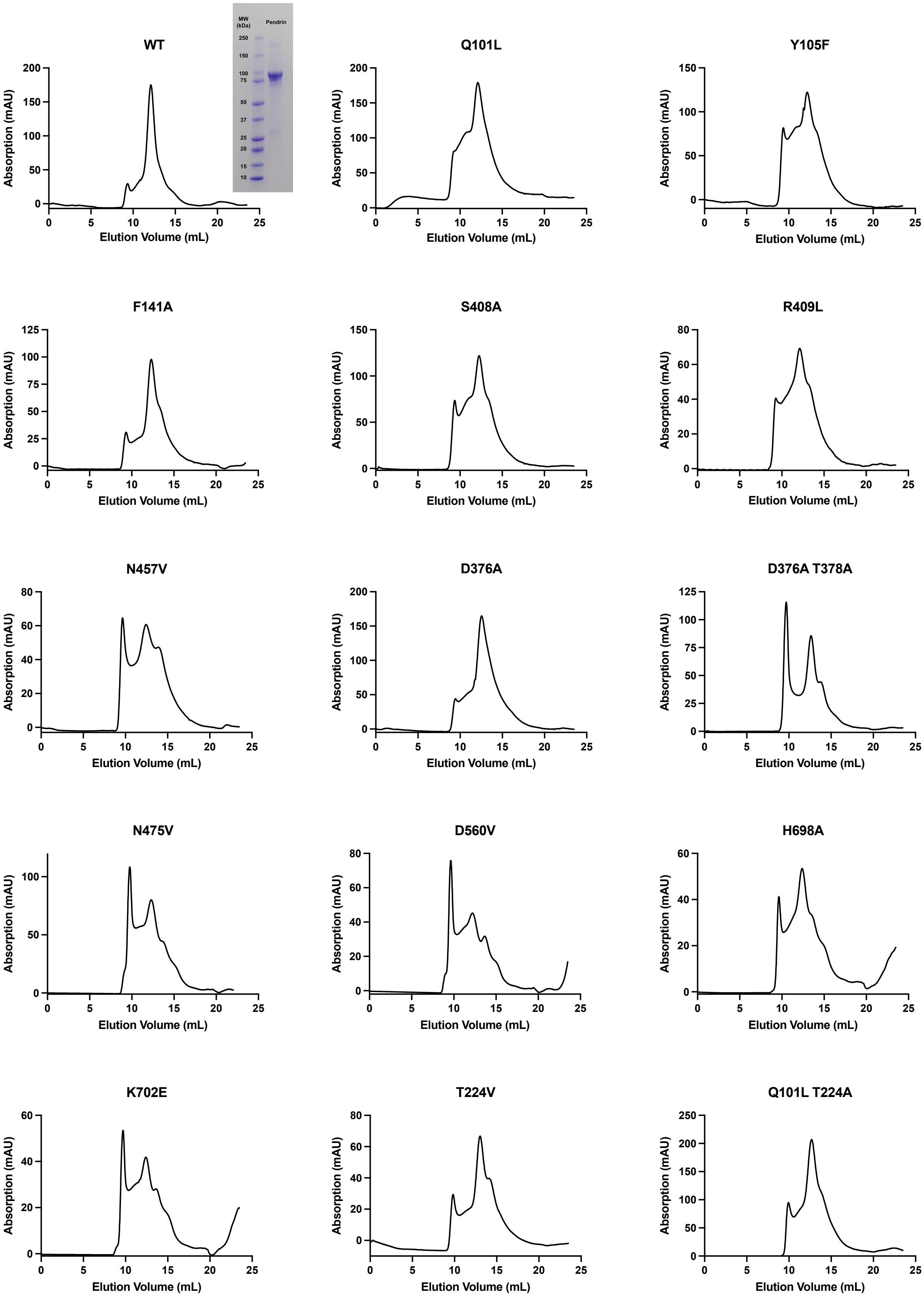
WT
MW (kDa)
Pendrin
250
150
100
75
50
37
25
20
15
10
Q101L
Y105F
F141A
S408A
R409L
N457V
D376A
D376A T378A
N475V
D560V
H698A
K702E
T224V
Q101L T224A
Absorption (mAU)
Elution Volume (mL)

Supplementary Figure 1. Purification profiles. Size-exclusion chromatography profiles of WT ssPendrin and mutants. The SDS-PAGE for WT ssPendrin is also shown.

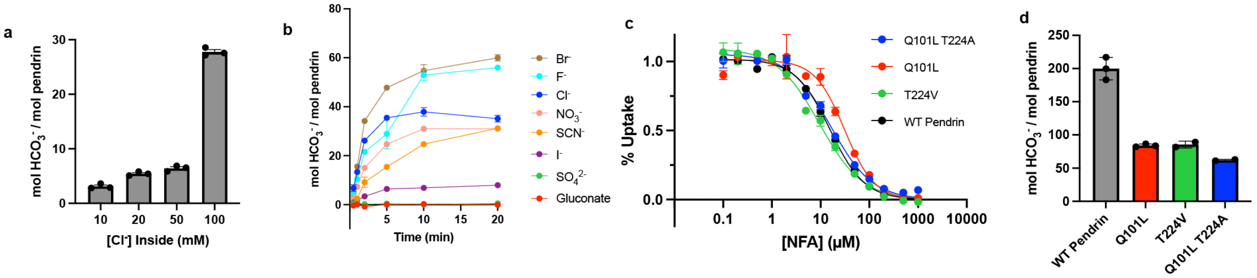

Supplementary Figure 2. Bicarbonate uptake and inhibition. a. Accumulation of 100 µM $^{14}C\text{-}HCO_3^-$ after 10 minutes with 1 mM Cl^- outside and varying concentrations of Cl^- inside of ssPendrin proteoliposomes. **b.** Time-dependent accumulation of 100 µM $^{14}C\text{-}HCO_3^-$ in ssPendrin proteoliposomes with 1 mM anion outside and 100 mM anion inside, with each anion shown in a different color as indicated. For all experiments, each data point is shown as mean ± s.e.m. (n≥3). Lines in Fig b simply connect the data points. **c.** Dose-response curve of NFA inhibition of 100 µM $^{14}C\text{-}HCO_3^-$ accumulation by WT (black), Q101L (red), T224V (green), and Q101L/T224A (blue) ssPendrin after 10 minutes, normalized as a percentage of the mean uptake with no NFA added. Data points were normalized and fit to the equation Y=Bottom + (Top-Bottom)/(1+(X/IC50)). Each data point is shown as mean ± s.e.m. (n≥2). **d.** Accumulation of 100 µM $^{14}C\text{-}HCO_3^-$ after 10 minutes with no NFA added.

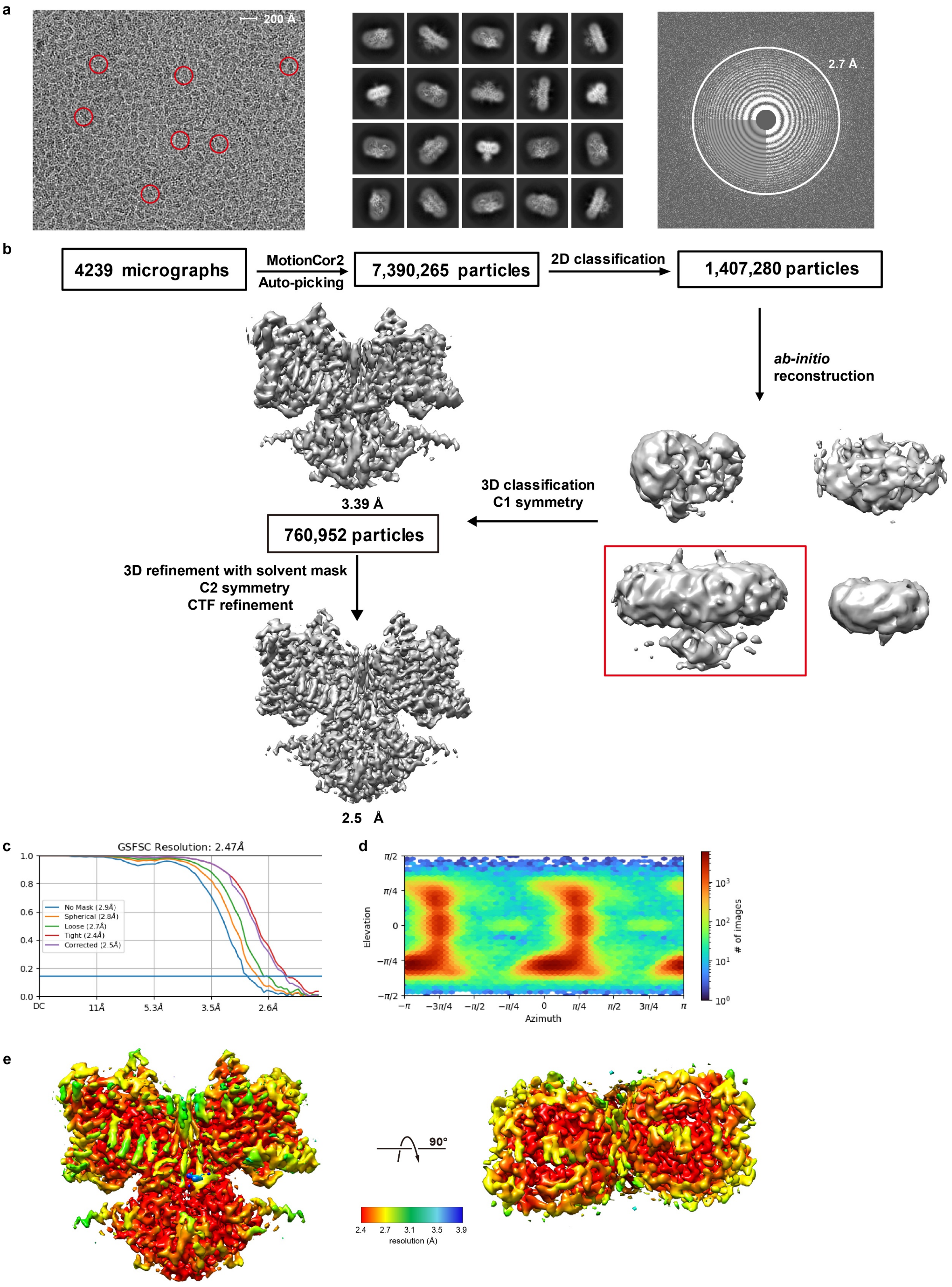

a
200 Å
2.7 Å
b
4239 micrographs
MotionCor2
Auto-picking
7,390,265 particles
2D classification
1,407,280 particles
ab-initio
reconstruction
3D classification
C1 symmetry
3.39 Å
760,952 particles
3D refinement with solvent mask
C2 symmetry
CTF refinement
2.5 Å
c
GSFSC Resolution: 2.47Å
No Mask (2.9Å)
Spherical (2.8Å)
Loose (2.7Å)
Tight (2.4Å)
Corrected (2.5Å)
DC
11Å
5.3Å
3.5Å
2.6Å
d
Elevation
Azimuth
# of images
e
90°
resolution (Å)
2.4
2.7
3.1
3.5
3.9

Supplementary Figure 3. Cryo-EM data processing of ssPendrin with Cl^-. a. A representative micrograph of ssPendrin with Cl^- (left), its Fourier transform (right) and representative 2D class averages (middle). Representative particles are highlighted with red circles. **b.** Flow chart for data processing and the final maps of ssPendrin with Cl^- (**Methods**). **c.** Gold-standard Fourier shell correlation (FSC) curve for the final map shown in **b**. **d.** Angular distribution of particles used in the final reconstruction. **e.** Local-resolution map of ssPendrin with Cl^- shown in two orientations.

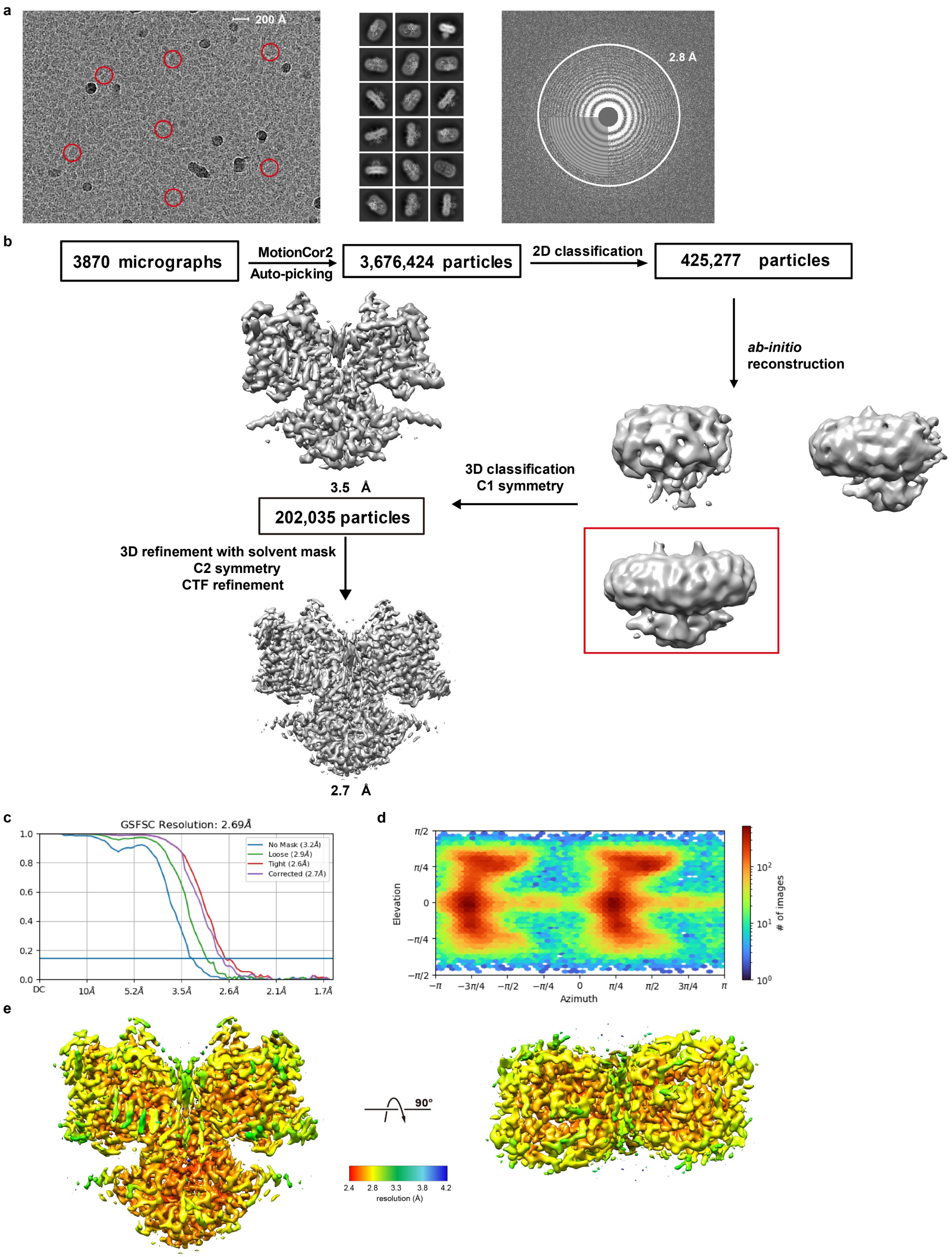

a
200 Å
2.8 Å
b
3870 micrographs
MotionCor2
Auto-picking
3,676,424 particles
2D classification
425,277 particles
ab-initio
reconstruction
3D classification
C1 symmetry
3.5 Å
202,035 particles
3D refinement with solvent mask
C2 symmetry
CTF refinement
2.7 Å
c
GSFSC Resolution: 2.69Å
No Mask (3.2Å)
Loose (2.9Å)
Tight (2.6Å)
Corrected (2.7Å)
1.0
0.8
0.6
0.4
0.2
0.0
DC
10Å
5.2Å
3.5Å
2.6Å
2.1Å
1.7Å
d
Elevation
Azimuth
π/2
π/4
0
−π/4
−π/2
−π
−3π/4
−π/2
−π/4
0
π/4
π/2
3π/4
π
# of images
10^2
10^1
10^0
e
90°
2.4
2.8
3.3
3.8
4.2
resolution (Å)

Supplementary Figure 4. Cryo-EM data processing of ssPendrin with HCO_3^-. a. A representative micrograph of ssPendrin with HCO_3^- (left), its Fourier transform (right) and representative 2D class averages (middle). Representative particles are highlighted with red circles. **b.** Flow chart for data processing and the final maps of ssPendrin with HCO_3^- (**Methods**). **c.** Gold-standard Fourier shell correlation (FSC) curve for the final map shown in **b**. **d.** Angular distribution of particles used in the final reconstruction. **e.** Local-resolution map of ssPendrin with HCO_3^- shown in two orientations.

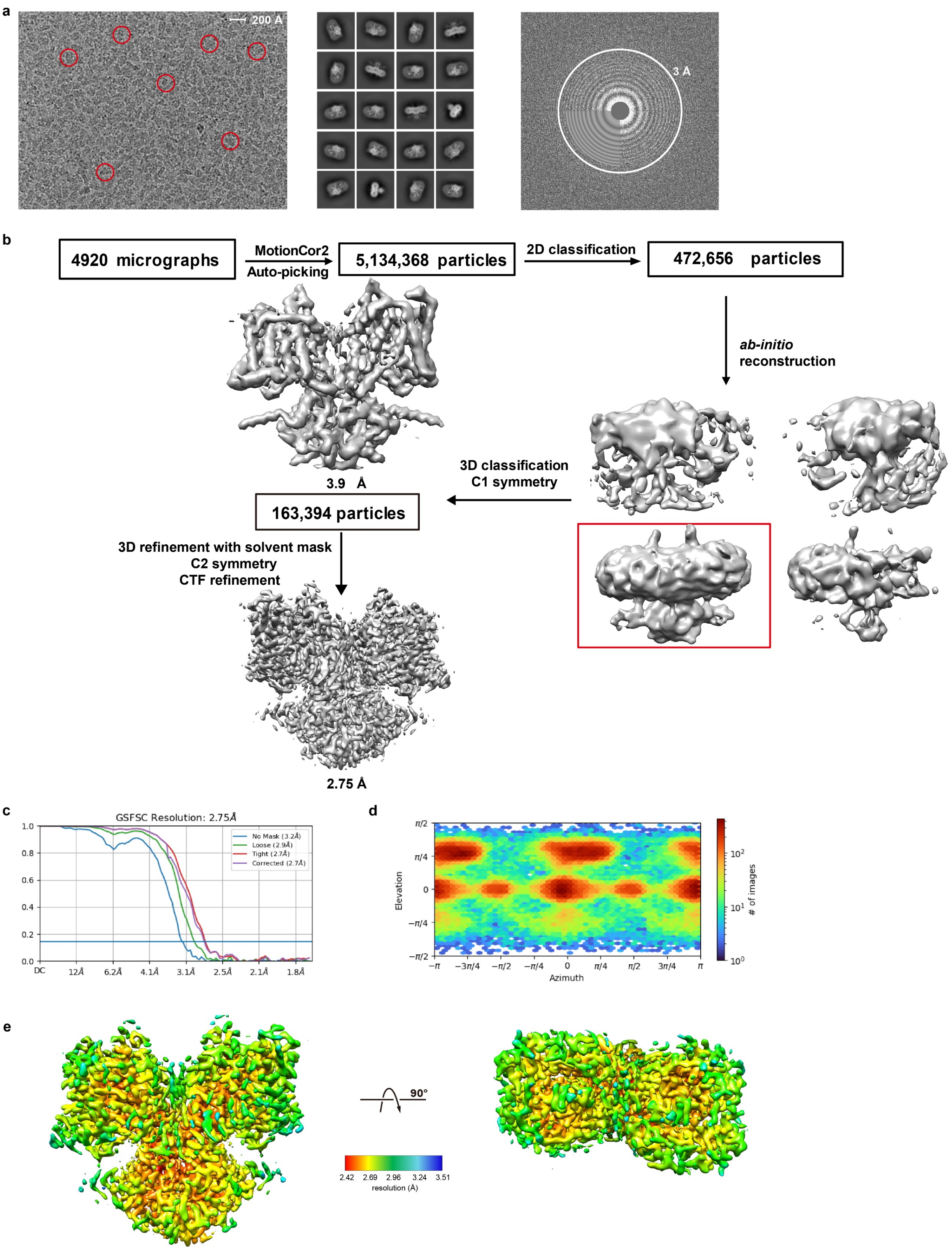
a
200 Å
3 Å
b
4920 micrographs
MotionCor2
Auto-picking
5,134,368 particles
2D classification
472,656 particles
ab-initio reconstruction
3D classification
C1 symmetry
163,394 particles
3.9 Å
3D refinement with solvent mask
C2 symmetry
CTF refinement
2.75 Å
c
GSFSC Resolution: 2.75Å
No Mask (3.2Å)
Loose (2.9Å)
Tight (2.7Å)
Corrected (2.7Å)
DC
12Å
6.2Å
4.1Å
3.1Å
2.5Å
2.1Å
1.8Å
d
Elevation
Azimuth
# of images
e
90°
2.42 2.69 2.96 3.24 3.51
resolution (Å)

Supplementary Figure 5. Cryo-EM data processing of ssPendrin with I^-. a. A representative micrograph of ssPendrin with I^- (left), its Fourier transform (right) and representative 2D class averages (middle). Representative particles are highlighted with red circles. **b.** Flow chart for data processing and the final maps of ssPendrin with I^- (**Methods**). **c.** Gold-standard Fourier shell correlation (FSC) curve for the final map shown in **b**. **d.** Angular distribution of particles used in the final reconstruction. **e.** Local-resolution map of ssPendrin with I^- shown in two orientations.

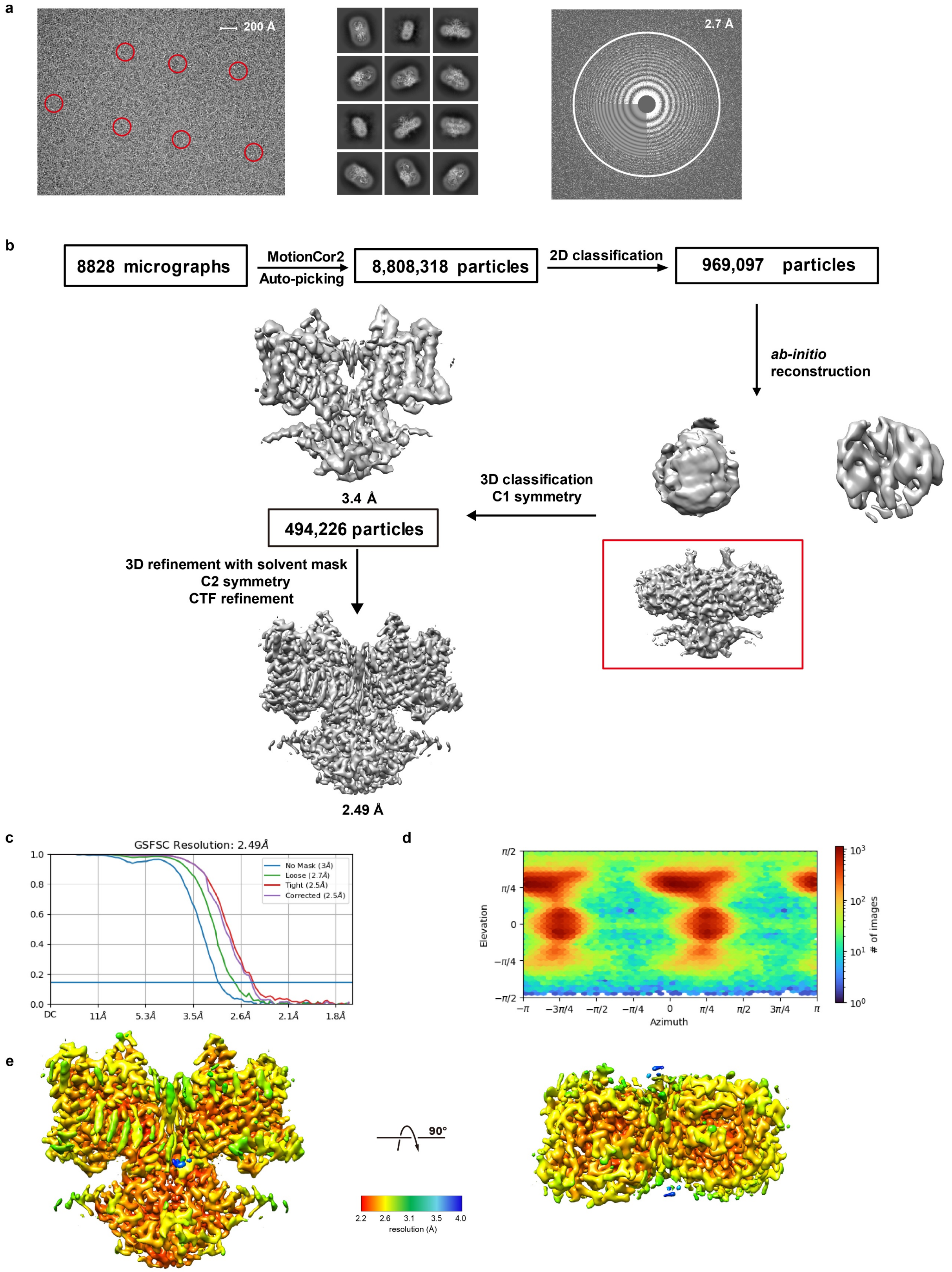
a
200 Å
2.7 Å
b
8828 micrographs
MotionCor2
Auto-picking
8,808,318 particles
2D classification
969,097 particles
ab-initio
reconstruction
3D classification
C1 symmetry
3.4 Å
494,226 particles
3D refinement with solvent mask
C2 symmetry
CTF refinement
2.49 Å
c
GSFSC Resolution: 2.49Å
No Mask (3Å)
Loose (2.7Å)
Tight (2.5Å)
Corrected (2.5Å)
DC
11Å
5.3Å
3.5Å
2.6Å
2.1Å
1.8Å
d
Elevation
Azimuth
# of images
e
90°
2.2 2.6 3.1 3.5 4.0
resolution (Å)

Supplementary Figure 6. Cryo-EM data processing of ssPendrin in apo. a. A representative micrograph of ssPendrin with I^- (left), its Fourier transform (right) and representative 2D class averages (middle). Representative particles are highlighted with red circles. **b.** Flow chart for data processing and the final maps of ssPendrin with I^- (**Methods**). **c.** Gold-standard Fourier shell correlation (FSC) curve for the final map shown in **b**. **d.** Angular distribution of particles used in the final reconstruction. **e.** Local-resolution map of ssPendrin with I^- shown in two orientations.

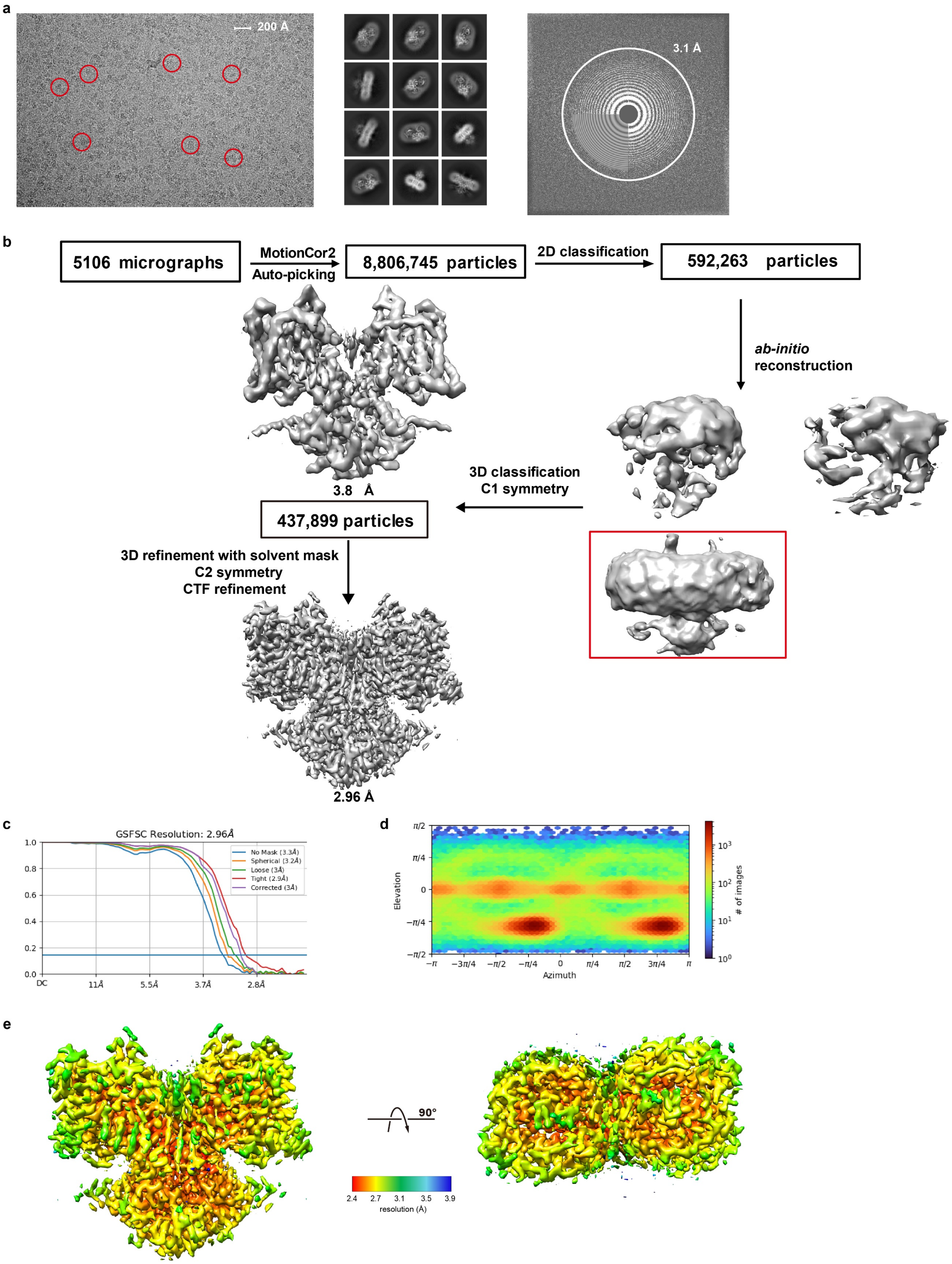
a
200 Å
3.1 Å
b
5106 micrographs
MotionCor2
Auto-picking
8,806,745 particles
2D classification
592,263 particles
ab-initio
reconstruction
3D classification
C1 symmetry
3.8 Å
437,899 particles
3D refinement with solvent mask
C2 symmetry
CTF refinement
2.96 Å
c
GSFSC Resolution: 2.96Å
No Mask (3.3Å)
Spherical (3.2Å)
Loose (3Å)
Tight (2.9Å)
Corrected (3Å)
DC
11Å
5.5Å
3.7Å
2.8Å
d
Elevation
Azimuth
# of images
e
90°
resolution (Å)

Supplementary Figure 7. Cryo-EM data processing of ssPendrin with Cl^- and NFA. a. A representative micrograph of ssPendrin with Cl^- and NFA (left), its Fourier transform (right) and representative 2D class averages (middle). Representative particles are highlighted with red circles. **b.** Flow chart for data processing and the final maps of ssPendrin with Cl^- and NFA (**Methods**). **c.** Gold-standard Fourier shell correlation (FSC) curve for the final map shown in **b**. **d.** Angular distribution of particles used in the final reconstruction. **e.** Local-resolution map of ssPendrin with Cl^- and NFA shown in two orientations.

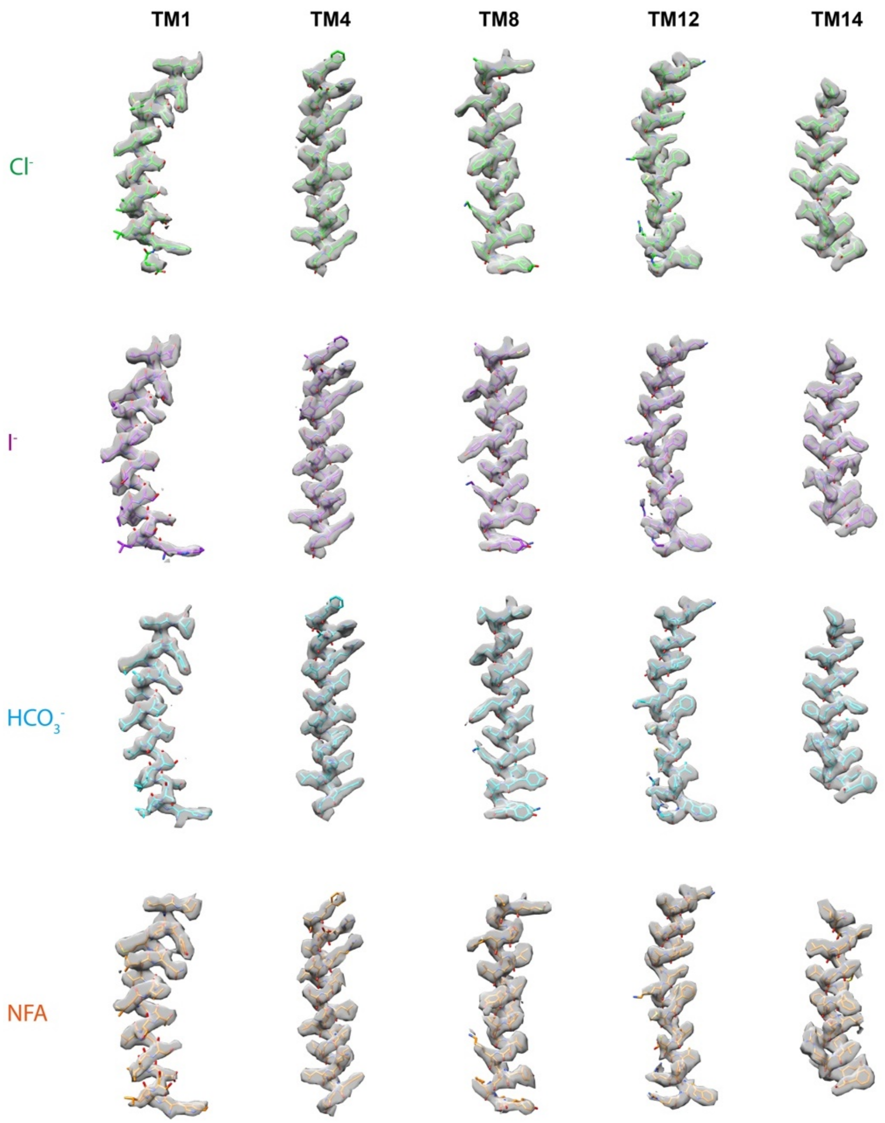

Supplementary Figure 8. Representative densities. Individual secondary structures of ssPendrin (sticks) and their densities (grey mesh) for TM1, TM4, TM8, TM12, and TM14. ssPendrin with Cl^- is shown in green, ssPendrin with I^- in magenta, ssPendrin with HCO_3^- in cyan, and ssPendrin with NFA in orange.

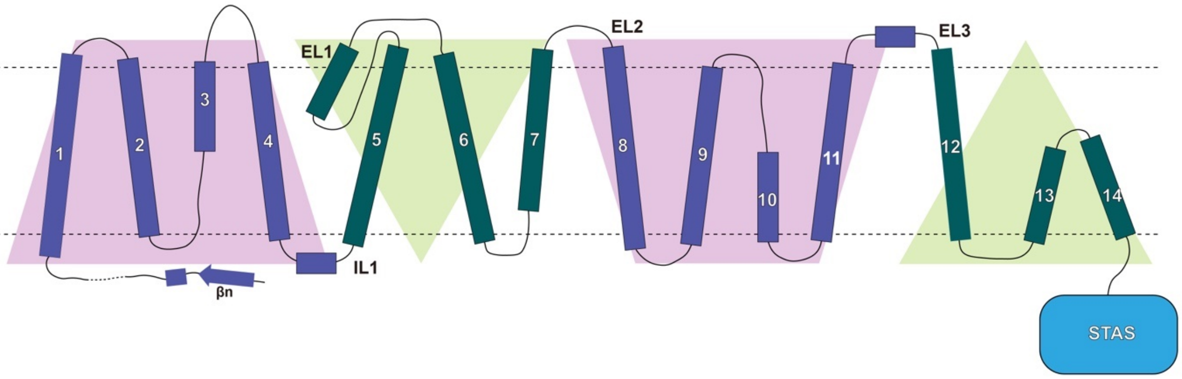

Supplementary Figure 9. Pseudo 2-fold symmetry of ssPendrin TM helices. Purple trapezoids represent the TM helices that form the transport domain, and green triangles indicate those forming the scaffold domain.

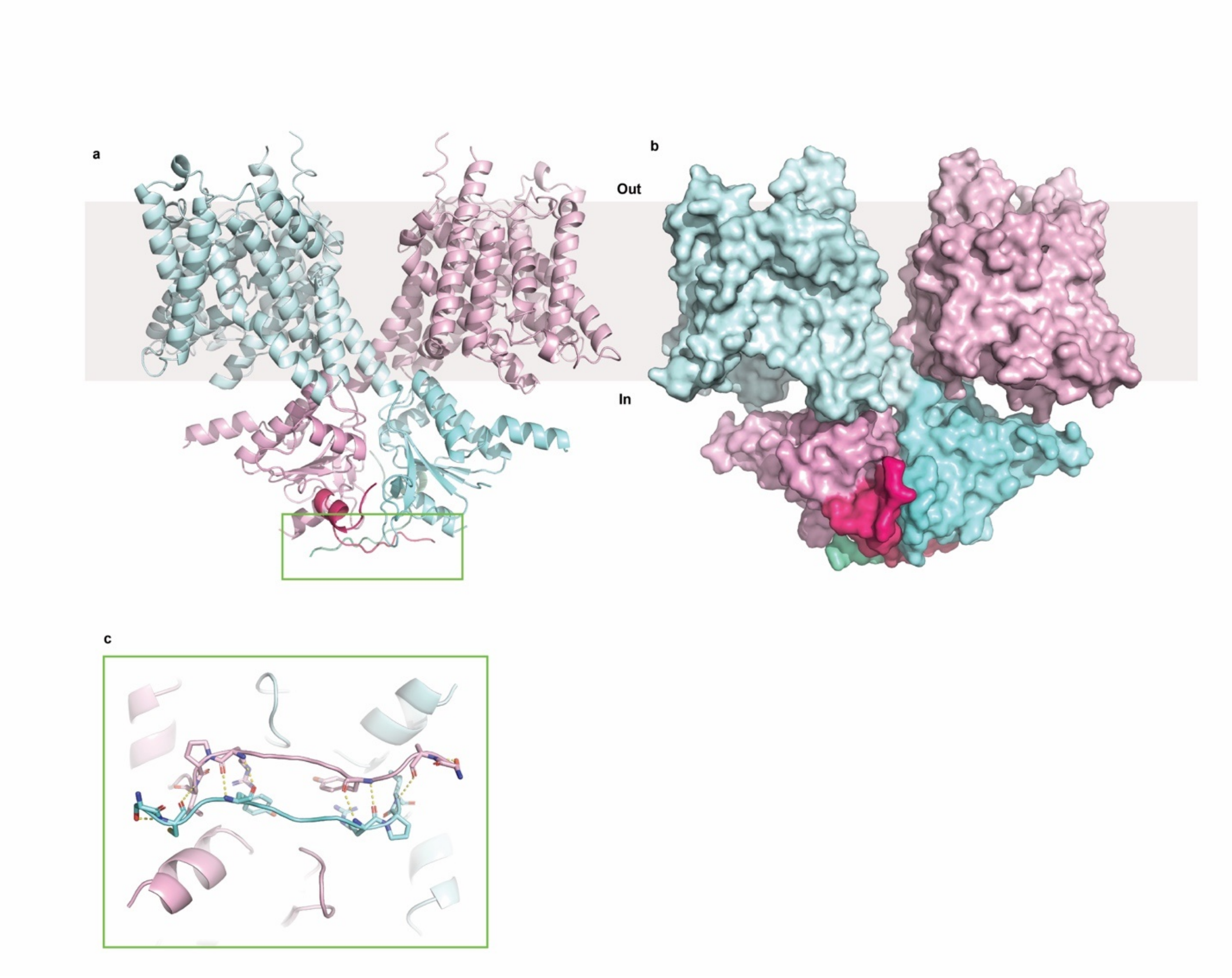

Supplementary Figure 10. Dimer interface. a and b. Structure of ssPendrin with the protomers colored in cyan and pink in cartoon (**a)** or surface representations (**b)**. Residues 17 to 39 from the N-termini are shown in darker blue and pink. **c.** The N-termini of the two protomers shown in sticks.

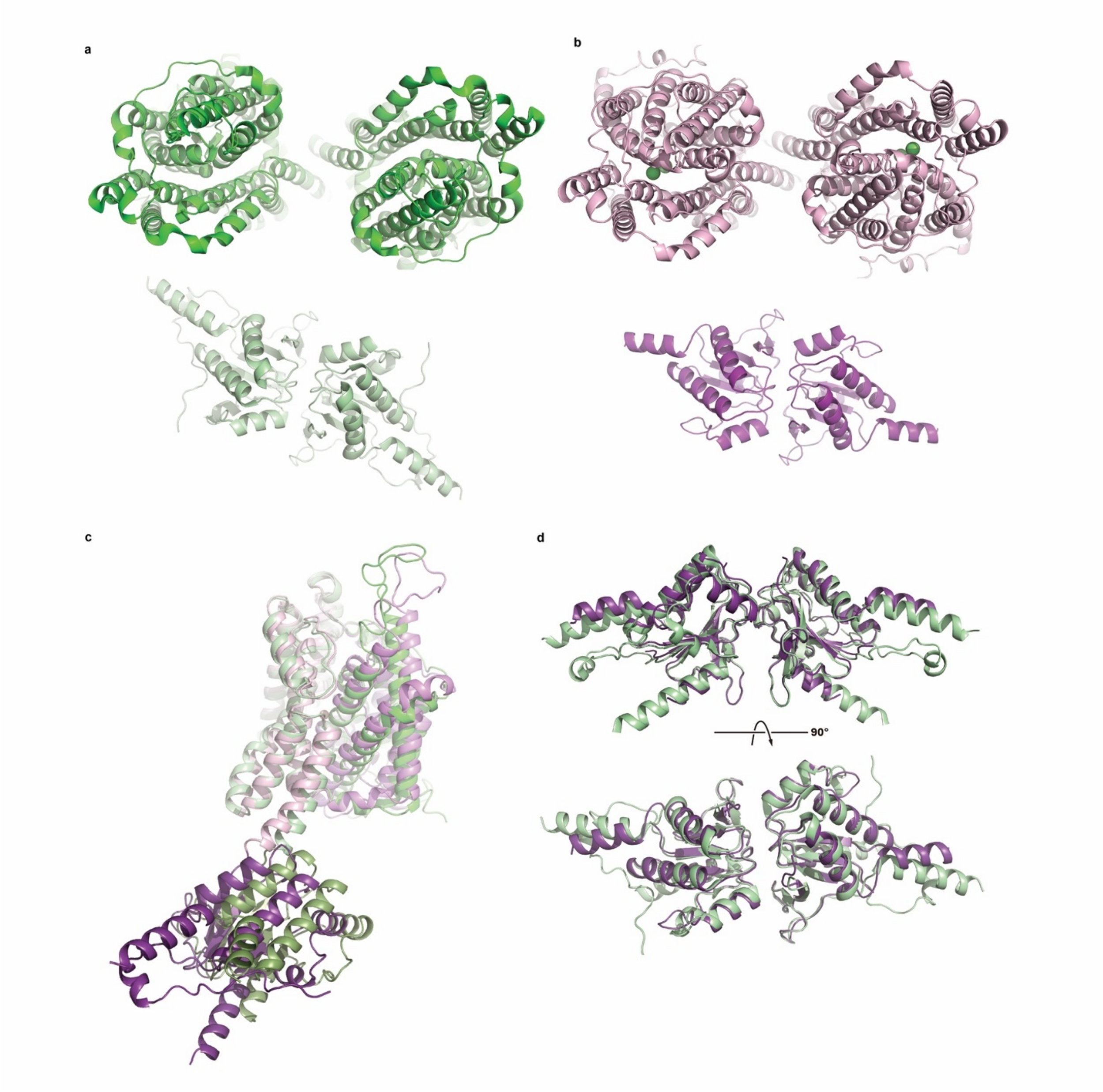

Supplementary Figure 11. Structural alignment of ssPendrin with Cl^{-} and prestin with Cl^{-}. a,b. Top and bottom view of ssPendrin with Cl^{-} (magenta) and prestin with Cl^{-} (green) (PDB ID: 7LGU [https://doi.org/10.2210/pdb7LGU/pdb])[1] dimers. **c.** View of the two monomer structures, aligned by their scaffold domains. **d.** Structural alignment of the two structures by their STAS domains, shown in two orientations.

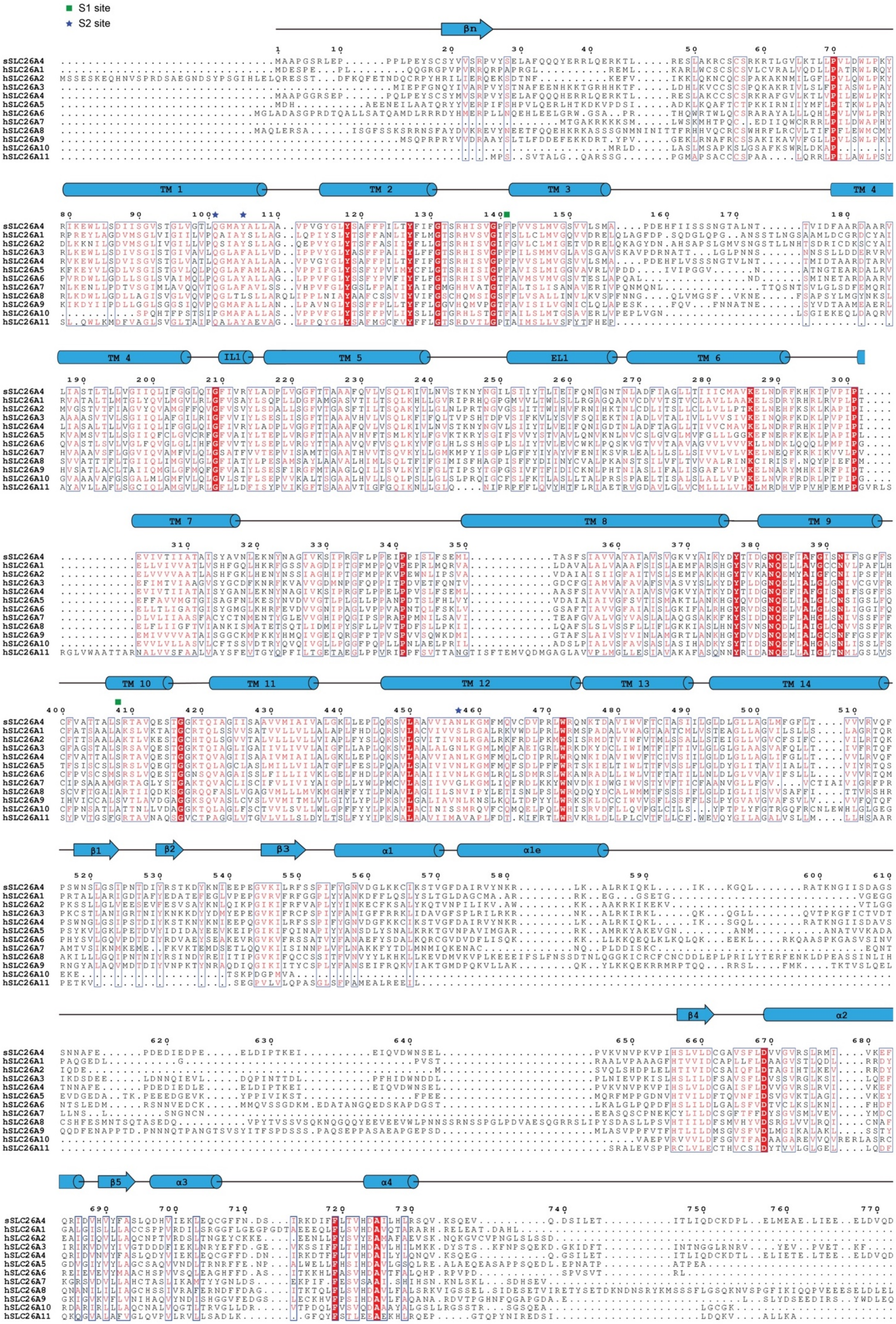

S1 site
S2 site
βn
TM 1
TM 2
TM 3
TM 4
IL1
TM 5
EL1
TM 6
TM 7
TM 8
TM 9
TM 10
TM 11
TM 12
TM 13
TM 14
β1
β2
β3
α1
α1e
β4
α2
β5
α3
α4
sSLC26A4
hSLC26A1
hSLC26A2
hSLC26A3
hSLC26A4
hSLC26A5
hSLC26A6
hSLC26A7
hSLC26A8
hSLC26A9
hSLC26A10
hSLC26A11

Supplementary Figure 12. Amino acid sequence alignment of ssPendrin and human SLC26 homologs. Amino acid sequences of sSLC26A4 (RefSeq accession XP_003357559 [https://www.ncbi.nlm.nih.gov/protein/XP_003357559.1]) , hSLC26A1 (RefSeq accession NP_071325 [https://www.ncbi.nlm.nih.gov/protein/NP_071325.2]), hSLC26A2 (RefSeq accession NP_000103 [https://www.ncbi.nlm.nih.gov/protein/NP_000103.2]), hSLC26A3 (RefSeq accession NP_000102 [https://www.ncbi.nlm.nih.gov/protein/NP_000102.1]), hSLC26A4 (RefSeq accession NP_000432 [https://www.ncbi.nlm.nih.gov/protein/NP_000432.1]), hSLC26A5 (RefSeq accession NP_001161434 [https://www.ncbi.nlm.nih.gov/protein/NP_001161434.1]), hSLC26A6 (RefSeq accession NP_001035544 [https://www.ncbi.nlm.nih.gov/protein/NP_001035544.1]), hSLC26A7 (RefSeq accession NP_001269285 [https://www.ncbi.nlm.nih.gov/protein/NP_001269285.1]), hSLC26A8 (RefSeq accession NP_001180405 [https://www.ncbi.nlm.nih.gov/protein/NP_001180405.1]), hSLC26A9 (RefSeq accession NP_443166 [https://www.ncbi.nlm.nih.gov/protein/NP_443166.1]), hSLC26A10 (RefSeq accession NP_597996 [https://www.ncbi.nlm.nih.gov/protein/NP_597996.2?report=genpept]), hSLC26A11 (RefSeq accession NP_001159819 [https://www.ncbi.nlm.nih.gov/protein/NP_001159819.1]) are aligned using the Clustal Omega Server[2,3]. Residues with red background are invariable, ones with red letters are highly conserved, and ones in blue boxes show similar residues. Secondary structures are marked on the top of the sequences.

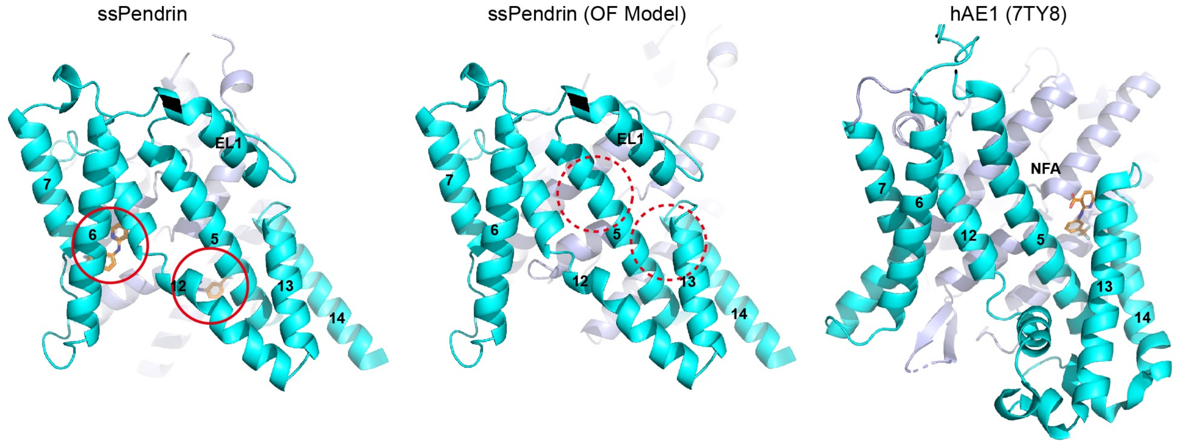

Supplementary Figure 13. Comparison of ssPendrin to hAE1 with NFA. (Left) The inward-facing structure of ssPendrin, (center) the model of outward-facing ssPendrin based on alignment with hAE1 (PDB: 7TY8 [https://doi.org/10.2210/pdb7TY8/pdb])[4], and (right) the outward-facing structure of hAE1 with NFA. The scaffold domain is colored in cyan and transport domain in light purple. Helices of the scaffold domain are labelled, and NFA is shown in orange. Red circles indicate NFA1 and NFA2 binding sites, and dashed red circles show their locations in the outward-facing ssPendrin model.

Supplementary Table 1. Summary of cryo-EM data collection, processing, and structural refinement

	Pendrin-Cl^- (EMD-40470) (PDB 8SGW)	Pendrin-I^- (EMD- 40479) (PDB 8SH3)	Pendrin-HCO_3^- (EMD-40507) (PDB 8SIE)	Pendrin-apo (EMD-42588) (PDB 8UUK)	Pendrin-NFA (EMD-40483) (PDB 8SHC)
**Data collection and processing**					
Magnification	81,000	105,000	105,000	105,000	81,000
Voltage (kV)	300	300	300	300	300
Electron exposure (e–/Å^2)	50	50	50	50	50
Defocus range (μm)	[-2.0, -0.8]	[-2.0, -0.8]	[-2.0, -0.8]	[-2.0, -0.8]	[-2.0, -0.8]
Pixel size (Å)	1.06	0.826	0.832	0.825	1.1
Symmetry imposed	C2	C2	C2	C2	C2
Initial particle images (no.)	7,390,265	5,134,368	3,676,424	8,808,318	8,806,745
Final particle images (no.)	760,950	163,394	202,035	494,226	437,899
Map resolution (Å)	2.5	2.8	2.7	2.5	3.0
FSC threshold	0.143	0.143	0.143	0.143	0.143
Map resolution range (Å)	2.3-3.9	2.4-4.0	2.4-4.2	2.2-4.0	2.4-3.9
**Refinement**					
Initial model used (PDB code)	None	8SGW	8SGW	8SGW	8SGW
Model resolution (Å)	2.8	3.0	2.9	2.9	3.1
FSC threshold	0.5	0.5	0.5	0.5	0.5
Model resolution range (Å)	2.6-3.0	2.8-3.1	2.7-2.9	2.7-3.1	2.9-3.2
Map sharpening *B* factor (Å^2)	-100	-114	-123	-110	-168
Model composition					
Non-hydrogen atoms	10,414	10,414	10,426	10,410	10,492
Protein residues	1,226	1,226	1,226	1,226	1,226
Ligands	38	38	38	34	40
B factors (Å^2)					
Protein	35.5	56.3	47.5	42.1	29.4
Ligand	24.8	64.5	55.7	81.9	37.6
R.m.s. deviations					
Bond lengths (Å)	0.003	0.003	0.003	0.003	0.003
Bond angles (°)	0.578	0.541	0.526	0.591	0.639
Validation					
MolProbity score	1.79	1.91	1.55	2.03	1.75
Clashscore	12.4	10.1	5.5	10.8	6.51
Poor rotamers (%)	0	0	0	0	0
Ramachandran plot					
Favored (%)	95.0	95.2	96.3	95.5	94.1
Allowed (%)	5.0	4.8	3.7	4.5	5.9
Disallowed (%)	0	0	0	0	0

References

1. Ge, A. J., Elferich, J., Dehghani-ghahnaviyeh, S. & Zhao, Z. Molecular mechanism of prestin electromotive signal amplification. *Cell* 1–26 (2021) doi:10.1016/j.cell.2021.07.034.
2. Madeira, F. *et al.* Search and sequence analysis tools services from EMBL-EBI in 2022. *Nucleic Acids Res.* **50**, W276–W279 (2022).
3. Robert, X. & Gouet, P. Deciphering key features in protein structures with the new ENDscript server. *Nucleic Acids Res.* **42**, 320–324 (2014).
4. Capper, M. J. *et al.* Substrate binding and inhibition of the anion exchanger 1 transporter. *Nat. Struct. Mol. Biol.* (2023) doi:10.1038/s41594-023-01085-6.
